# Research Progress and Application Prospects of Gel Electrolytes

**DOI:** 10.3390/gels11121002

**Published:** 2025-12-12

**Authors:** Andrea Zille, Ana Isabel Ribeiro

**Affiliations:** 2 C2T—Centre for Textile Science and Technology, Campus Azurém, University of Minho, 4800-058 Guimarães, Portugal; a.isabel.f.ribeiro@gmail.com

## 1. Introduction

Gel electrolytes have rapidly emerged as indispensable components in modern electrochemical systems, bridging the performance gap between liquid and solid electrolytes. These soft, ion-conducting materials combine the high ionic conductivity of liquids with the mechanical stability of solids, offering unique advantages in a single platform. In energy storage, gel polymer electrolytes (GPEs) enable safer, high-energy density batteries by mitigating the leakage, flammability, and dendrite formation issues that plague traditional liquid electrolytes. Their inherent flexibility and viscoelasticity also make them ideal for flexible and wearable electronics, where they can sustain mechanical deformation while maintaining ionic conduction. Indeed, applications now span from lithium batteries and supercapacitors to stretchable sensors and bioelectronic devices, underscoring the growing importance of gel electrolytes in delivering safe, high-performance energy storage and conversion systems.

Recognizing these trends, the first edition of this Special Issue, “Research Progress and Application Prospects of Gel Electrolytes”, was conceived to highlight cutting-edge research in this vibrant field. That Special Issue attracted ten contributions encompassing a diverse range of topics, from advanced materials development to device integration. The works collectively reinforced how gel electrolytes are enabling breakthroughs in electrochemical technologies while also shedding light on remaining challenges. In the following paragraphs, we briefly summarize the key results and insights from the first edition, which form a foundation for the continued exploration in this second edition.

Realizing these patterns, the idea of the first volume of this Special Issue, “Research Progress and Application Prospects of Gel Electrolytes”, was born to emphasize the forefront research of this lively sector. This edition attracted ten submissions covering a wide range of topics, starting from the development of advanced materials to device integration. The papers, as a whole, emphasized the role of gel electrolytes in enabling the breakthrough of electrochemical technologies, while at the same time, they pointed to the issues that persist. Next, we briefly identify the main findings and insights of the first volume that serve as a stepping stone for more in-depth research in this second volume.

## 2. Highlights from the First Edition

https://www.mdpi.com/journal/gels/special_issues/4638YD96UE (accessed on 28 November 2025).

**Advances in Energy Storage:** The first edition was largely dedicated to gel electrolytes for batteries and supercapacitors [contributions 1,2]. One of the primary themes was how material design can not only increase the performance of a battery but also make it safer. For instance, Mao et al. fabricated a composite gel electrolyte by incorporating a metal–organic framework (ZIF-8) in a UV-cured polymer matrix, which had an ionic conductivity of ~1.17 × 10^−3^ S/cm and a long electrochemical stability window of 4.7 V [contribution 3]. Consequently, this MOF-derived gel made it possible to achieve high specific capacities and enhanced rate capability in lithium metal cells, thus a better cycling performance was obtained. In their study, Yao et al. similarly prepared a UV-crosslinked PVDF-HFP/PAN hybrid gel electrolyte with LLZTO ceramic nanoparticles and achieved an ionic conductivity at room temperature of 2.8 × 10^−4^ S/cm [contribution 4]. It is worth noting that a LiFePO_4_||Li cell with this gel gave ~164 mAh·g^−1^ at 0.1 C, and the capacity after 200 cycles was ≈89%, which represents a remarkable degree of short-term stability in solid-state cells. This indicates how far researchers have come in developing gel electrolytes that are capable of fast ion transport and stable long-term operation. Alongside these findings, scientists also face the problem of high interfacial resistance in multi-layer cell architectures. Chen et al. looked into the design of a multi-layer solid-state battery and found that the capacity in the stacked cells decreased much more rapidly compared with the single-layer cells. Three-layer stack had only 15.6% capacity retention after 100 cycles vs. single-layer cell (∼48.9% retention) [contribution 5]. This discovery exemplifies how the improvement of interfaces and architectures is indispensable as gel-based batteries are becoming thicker or of higher capacity.

**Flexible Electronics and Sensors:** One of the most notable changes for electronic devices due to gel electrolytes is the complete revolution of their form factors. Ribeiro et al., in their comprehensive review, examined the role of gel electrolytes in the development of textile-based power sources and wearable devices. Gels can be easily integrated into fibers, yarns, and fabric and thus not only enhance safety (no liquid leakage), but also mechanical flexibility and conformability, which are indispensable properties for powering wearable devices used in healthcare, sports, and IoT applications [contribution 6]. The review also states that gel electrolytes provide leak resistance and improved interface compatibility in such systems; however, there are still difficulties in ionic conductivity and stability over long periods under repeated deformation. The first volume of the Special Issue showed that the use of gels is spreading beyond energy storage to the field of sensing technologies. For example, Cedeño Mata et al. developed a polyvinyl alcohol-based ionic liquid gel polymer electrolyte (ILGPE) with the addition of SiO_2_ nanoparticles and PVP to improve the performance of a capacitive humidity sensor [contribution 7]. The composite gel resulted in a significantly improved sensor response and hysteresis less than 0.02 over 20–90% relative humidity due to the designed pore structures and the interactions between the SiO_2_/PVP fillers and the host matrix. This is a perfect example of how gel electrolytes can be reconciled with the functional needs (here water uptake dynamics) of sensor applications. These advancements in flexible and functional gels are indicative of an upcoming era in which power sources and sensors can be embedded more safely and intimately in wearable, portable, or biologically integrated devices.

**Innovations in Fabrication and Design:** Several works in the first edition led to the exploration of new fabrication methods for gel electrolytes and greater understanding, showing that the field is maturing and uses a variety of interdisciplinary tools. Yao et al. reported on the employment of 3D printing (direct ink writing) in the fabrication of a “ceramic-in-gel” electrolyte with a highly controlled porous microstructure [contribution 8]. The constructed gel revealed a standard sponge-like structure and reached an ionic conductivity of ~5.77 × 10^−4^ S/cm, which corresponds to a substantial improvement of Li-ion transport at room temperature. This innovative manufacturing method not only facilitates the design of electrolyte shapes (e.g., complex geometries or thin-film designs) for better contact in batteries and microdevices but also allows for the exploration of other possibilities in the field of electrolytes. On the other hand, data-driven methods are employed to decipher gels’ complex chemistry. Xu et al. presented an interpretable machine learning framework that links PVA hydrogel formulation parameters to mechanical performance [contributions 9,10]. Using large-scale data point examination, their model speculated that PVA molecular weight was the main factor controlling the tensile properties, while the degree of hydrolysis and cross-linker content played the next most important roles in the hierarchy. Moreover, the insights gained through SHAP interpretability analysis offer a solid and understandable platform for designing the next generation of hydrogels with desirable properties. In parallel, another paper approached the topic in a different way, introducing machine learning for supercapacitors’ hydrogel electrolyte optimization and revealing design factors leading to better capacitance and cyclability (e.g., polymer composition, crosslink, and density). In the end, both advanced fabrication and computational modeling methods spearhead the transformation of gel electrolytes from a trial-and-error approach toward *engineered* gels with predictable structure–property relationships.

The first volume of this Special Issue has been a splendid demonstration of gel electrolytes’ plasticity and their ripple effect across numerous fields. The array of contributions ranges from high-energy batteries and flexible supercapacitors to wearable power textiles and sensors and underlines the fact that gel-based electrolytes are driving the next generation of electrochemical technologies. In addition to this, scientists have unveiled several tangible problems, e.g., how to reach a high ionic conductivity at room temperature, ensure interfacial stability in full cells, and produce at a large scale in a sustainable manner, that still need to be solved before gels can be fully applied. This set of discoveries heralds the second edition, which is intended to comprehend the progress made and further advance the field.

## 3. Conclusions and Outlook

The creation of gel electrolytes will expand rapidly in many innovative ways in the future. Advanced material designs that not only enhance ionic transport but also provide mechanical strength and safety are one of the main themes being discussed. New polymer matrices (e.g., bio-based or supramolecular polymers), inorganic–organic hybrid networks, and nanoengineered fillers that jointly create fast ion channels without compromising stability are among these designs. More specifically, the main design targets will be to increase Li^+^ transference number and lower interfacial resistance, especially in high-current applications.

Similarly, a lot of attention is being directed toward thermal and electrochemical stability enhancement in gels in order to create batteries that are safe under more extreme conditions. Recently, it has been proven in several experiments that, for example, blend-type gel membranes can last structurally at high temperatures and for long cycles. The invention of inherently non-flammable, self-extinguishing, or thermally adaptive gel electrolytes may not only enhance the safety of EVs but also that of grid storage, thus alleviating a substantial part of the safety concern.

Moreover, fostering sustainability and retaining a good performance over long periods of time is a great challenge for gel electrolytes. As the discipline develops, the scientific community aims to replace volatile or poisonous agents with environmentally friendly ones and simplify gel electrolyte production overall. The establishment of fluorine-free gel polymer electrolytes that generate a stable, LiF-free solid–electrolyte interphase is a milestone example of this effort. By using recyclable or bio-degradable polymer gels, water-based synthesis routes, and inexpensive dopants, the research community will be in a position to guarantee that gel electrolytes are a part of the solution to the energy issue.

In addition, durability over the long term under real-world conditions (humidity, temperature fluctuations, and prolonged cycling) will be the yardstick of success. An example of research into aging mechanisms is monitoring water uptake in hydrogel electrolytes or electrode/electrolyte interphase changes over hundreds of cycles, which will aid the development of gels capable of maintaining their performance for years of operation.

Above all, the goal of this work that links different research themes is the eventual incorporation of gel electrolytes into practical devices. This second edition of our Special Issue will be enriched with papers demonstrating the use of gels in the fabrication of batteries, supercapacitors, fuel cells, sensors, and other technologies. Such papers will illuminate application prospects where gel electrolytes can be used in a lithium metal pouch cell with greatly reduced dendrite growth, or a hydrogel film is used to enable a flexible bio-sensor. We are also very interested in new ionic transport mechanisms, such as assisted ion hopping along polymer segments or deliberately phase-separated pathways that imitate biological ion channels, which, in turn, may result in electrolytes that do not compromise conductivity, selectivity, and mechanical strength. By delving into ion dynamics at a very basic level (using sophisticated spectroscopy, modeling, and theory), researchers can develop gel electrolytes that are capable of transporting ions at higher rates and with greater selectivity than ever before.

By issuing this call for papers for a second edition of the Special Issue, we are setting the bar very high. We are looking to gather studies that do not merely present new material or performance benchmarks but also shed light on the scientific understanding of gel electrolyte operation and optimization. Ideally, each paper submitted should be a small contribution to the larger puzzle: through the intelligent use of gels, how can one create safe and efficient electrochemical systems with a long duration? We hope the concerted efforts recorded in this Special Issue (2nd Edition) will represent a departure from the familiar and, thus, provide a source of new ideas for both academic and industrial researchers. The future of gel electrolytes is very promising considering the fast-paced and interdisciplinary nature of this field, which covers polymer chemistry, materials science, electrochemistry, and device engineering. We believe that persistent investigations will result in gel-derived energy and electronics devices that not only perform well but are also flexible, dependable, and eco-friendly.

In conclusion, gel electrolytes have now moved to the forefront of technological innovation in energy storage and flexible electronics. The advances in the research highlighted in the first edition have made more bold developments possible. We, as the Guest Editors of this second edition, are excited about the new discoveries and ingenious solutions that will be disclosed. Collectively, these works will facilitate the move of gel electrolytes from experimental research to practical use—thus, enabling the advent of a new era of safe, high-performing electrochemical devices.

## Data Availability

No new data were created or analyzed in this study.

